# Supplementary material for: The rpoS gene confers resistance to low osmolarity conditions in Salmonella enterica serovar Typhi
Source: PLoS One. 2022 Dec 16;17(12):e0279372. doi: 10.1371/journal.pone.0279372 (PMC9757558; doi:10.1371/journal.pone.0279372)
Supplement: Attachment — Submitted filename: ResponseToReviewers.docx [file pone.0279372.s018.docx]

My co-authors and I would like to thank the editors and reviewers for their careful evaluation of our manuscript, and for their thoughtful comments and suggestions. We have carried out the key experiments suggested by the reviewers and have incorporated the results into the revised manuscript. The new findings have helped significantly to strengthen the overall conclusions of the study. We hope that the work will now be considered suitable for publication in *PLoS One*.

We have responded to each of the points mentioned by the reviewers as indicated below. The page and line numbers mentioned in the responses refer to the marked-up manuscript file.

**Reviewer 1**

We are gratified that the reviewer found the manuscript to be “well-written” and “a good start to an interesting study of the role of RpoS in resisting hypotonic conditions”.

Major

1. *Please provide more information in the manuscript about the plasmid pBH….*

In response to the reviewer’s request, we have now provided more information about pBH in the Methods section of the revised manuscript (p. 8, lines 236-239), including promoter (*trp-lac*) and copy number (high copy). Since the *S*. Typhi Ty2 strain does not express the *lac* repressor, the promoter functions in a constitutive fashion.

2. *Why did the authors choose to use constitutive expression for this study? The authors need to be careful about the conclusions and title because this study is solely testing whether constitutive expression of rpoS can confer resistance to low osmolarity.*

We chose pBH-mediated constitutive expression of *rpoS* because our earlier work with this plasmid yielded robust and reliable expression in *Salmonella* (*Infect. Immun.* 2000; 68: 5567). This has been explained on p. 8, lines 238-239 of the revised manuscript. The reviewer raises a valid concern that the results with this system as reported in the original version of the manuscript merely show that constitutive expression of *rpoS* can confer resistance to hypotonic conditions. However, we have now carried out additional experiments using *S*. Typhi strains kindly provided by Dr. Roy Curtiss III that substantiate and extend our findings with plasmid-directed *rpoS* expression in Ty2. In these new experiments, we assessed the survival in distilled water of the strains χ3744 (*S*. Typhi ISP1820, wild-type *rpoS* gene), χ9061 (*S*. Typhi ISP1820, *rpoS* inactive allele expressing a truncated protein) and χ9066 (*S*. Typhi ISP1820, arabinose-inducible wild-type *rpoS*). As shown in Fig. 7 and described on p. 15-16, lines 451-472 of the revised manuscript, the viability of χ3744 was not compromised by incubation in water, in contrast to the *rpoS*-mutant strain Ty2. The *rpoS*-inactive strain χ9061 had significantly reduced viability in water, an abnormality that was corrected in χ9066 only when the bacteria were grown in the presence of arabinose. These new findings confirm the overall conclusions of the study and the appropriateness of the title.

3. *Please provide growth curves for strains containing empty plasmid and the rpoS expression plasmid…..*

To address the reviewer’s concerns, we have now provided growth curves for the parental Ty2 strain along with the strains containing empty plasmid (Ty2/pBH) and the *rpoS* expression plasmid (Ty2/rpoS) (Fig. 5A of the revised manuscript, described on p. 13, lines 387-388). The data indicate that there is no difference between the growth patterns of the latter 2 strains, although they both have a longer lag phase than the parental Ty2. Importantly, both of the plasmid-containing strains reach saturation at about the same time, including the time at which the cultures are harvested for analysis. Thus, it is unlikely that differences in growth phase at the time of analysis can explain the difference in susceptibility to hypotonic conditions between Ty2/pBH and Ty2/rpoS. Moreover, our new experiments with the *S*. Typhi strains from Dr. Curtiss clearly demonstrate that the viability of a strain with wild-type *rpoS* is not affected by incubation in distilled water, and that inactivation of *rpoS* in this strain significantly compromises viability under these conditions (Fig. 7 of the revised manuscript). These findings confirm the role of *rpoS* in resisting low osmolarity, and also indicate that our results with plasmid-directed expression of *rpoS* in Ty2 are unlikely to reflect the effects of over-expression or inappropriate expression.

4. *Please provide evidence for expression of rpoS in the strain containing the rpoS expression plasmid…..Evidence of rpoS gene expression by qRT-PCR (of rpoS itself and/or selected rpoS target genes)….is needed…*

To address the reviewer’s concern, we have carried out qRT-PCR analysis to examine expression of the *rpoS* target gene *katE*. The results (Fig. 5B of the revised manuscript, described on p. 13-14, lines 389-402) indicate that expression of *katE* is about 45-fold higher in the strain containing the *rpoS* expression plasmid (Ty2/pBHSTm*rpoS*) relative to the parental Ty2 strain. As requested by the reviewer, we also examined *katE* expression in an *S*. Typhi strain that has a wild-type *rpoS* gene (χ3744), and found it to be about 600-fold higher than in Ty2. Thus, the effect of plasmid-mediated *rpoS* expression on *katE* levels in Ty2 can be considered to be within the wild-type range.

Minor

1. *Please include additional information about tube size and volumes used for culture growth….*

Bacterial strains were grown at 37^o^C in sterile 12 ml polypropylene tubes, using 2 ml of LB in loosely capped tubes or 10 ml of LB in tightly capped tubes for shaking or static cultures, respectively. For generation of growth curves, shaking cultures were carried out in triplicate in sterile 24-well tissue culture plates with 1.2 ml of LB per well. 100 µl of the culture was removed from each well at each time point to measure absorbance in a microplate reader. To ensure that the culture volume in the wells was not depleted, separate plates were used for the measurements at 0-6 hr, 7-16 hr and 24-26 hr. As requested by the reviewer, we have now provided these details in the Methods section (p. 5, lines 132-139, and p. 6, lines 159-169 of the revised manuscript).

2. *Please provide more information about the qRT-PCR analysis….*

As requested by the reviewer, we have provided a more detailed description of the various steps of the qRT-PCR analysis, including temperatures and times of incubation, reaction component concentrations and amplication conditions (Methods section, p. 7, lines 190-212 of the revised manuscript). Sequences of primers were designed using Primer-BLAST and their specificity was confirmed by melting curve analysis of the amplification products. All products yielded single, sharp melting curve peaks.

3. *In Figure 5, why does the presence of the empty vector confer some resistance to hydrogen peroxide? Is this statistically significant?*

Even though the peroxide resistance of the strain with the empty vector appears to be greater than the parental Ty2 strain, the difference is not statistically significant and presumably reflects experimental variation. We have mentioned this in the revised legend to Fig. 5 (p. 15, line 433 of the revised manuscript).

**Reviewer 2**

We are pleased that the reviewer found the study to be “well-presented with clear logic flow and associated figures”. The specific comments made by the reviewer have been addressed below.

1. *The introduction needs more discussion of the rpoS gene….*

As requested by the reviewer, we have expanded the Introduction section of the manuscript to present more information about *Salmonella* *rpoS* and have cited the relevant papers, including key publications from the Curtiss and Norel labs, in both the Introduction and Discussion (p. 4-5, lines 94-115, and p. 17, lines 497-498 of the revised manuscript).

2. *Line 56-58. This sentence should be cited with references….*

We have cited the relevant references in support of this statement (p. 3, lines 65-67 of the revised manuscript).

3. *Sentence ending in line 61 should also have more references….*

We have supported this statement with additional references beyond the review originally cited (p. 3, lines 67-70 of the revised manuscript).

4. *The “nail in the coffin” for this study would have been to take a WT, low osmolarity resistant S. Typhi strain and mutate that rpoS gene…..*

Thank you for this suggestion. We have carried out the exact experiment proposed by the reviewer using *S*. Typhi strains kindly provided by Dr. Roy Curtiss III. In this new analysis, we assessed the survival in distilled water of the strains χ3744 (*S*. Typhi ISP1820, wild-type *rpoS* gene), χ9061 (*S*. Typhi ISP1820, *rpoS* inactive allele expressing a truncated protein) and χ9066 (*S*. Typhi ISP1820, arabinose-inducible wild-type *rpoS*). As shown in Fig. 7 and described on p. 15-16, lines 451-472 of the revised manuscript, the viability of χ3744 was not compromised by incubation in water, in contrast to the *rpoS*-mutant strain Ty2. The *rpoS*-inactive strain χ9061 had significantly reduced viability in water, an abnormality that was corrected in χ9066 only when the bacteria were grown in the presence of arabinose. These results are consistent with our findings with plasmid-directed expression of *rpoS* in Ty2, and confirm the idea that *rpoS* is required to resist low osmolarity conditions.

5. *The authors should more fully discuss the possibility that the low osmolarity sensitivity in Ty2 could be due to some other factor than rpoS…..*

Our new findings with the strains obtained from Dr. Curtiss (Fig. 7) indicate clearly that *rpoS* is necessary for normal survival under low osmolarity conditions. However, we agree with the reviewer that it is formally possible that the plasmid-directed expression of *rpoS* in Ty2 could compensate for some other factor that makes this strain sensitive to low osmolarity. We have mentioned this possibility on p. 15, lines 451-454 of the revised manuscript in the context of the new findings shown in Fig. 7.

*The reviewer also mentions that “it would be helpful to introduce the cloned rpoS into other S. Typhi mutant, low osmolarity sensitive strains” and that “it would be informative to test the S. Typhi strains/isolates with WT rpoS genes for low osmolarity resistance”.*

As mentioned above, we have tested the *S*. Typhi strain χ3744, which has a wild-type *rpoS* gene, and have shown that it is resistant to incubation under low osmolarity conditions (Fig. 7 of the revised manuscript). While we agree with the reviewer that it would also be helpful to express the cloned *rpoS* gene in other *S*. Typhi strains with mutant *rpoS*, this experiment is not easily done since we would first have to identify the mutant strains among multiple candidates, a process that would probably require sequencing the relevant genomic region. Moreover, the outcome of this effort would serve mainly to confirm our findings with Ty2. For these reasons, we respectfully submit that our results with *rpoS* expression in Ty2, together with the new findings generated using the *rpoS* wild-type, *rpoS* inactivated and *rpoS* inducible strains (Fig. 7), already provide strong evidence that *rpoS* plays an important role in resistance to low osmolarity. Thus, we feel that expression of *rpoS* in additional *rpoS* mutant strains is not essential to the scope of the present work.
